# Factors determining membership in community-based health insurance in West Africa: a scoping review

**DOI:** 10.1186/s41256-022-00278-8

**Published:** 2022-11-28

**Authors:** Kaba Kanko Conde, Aboubacar Mariama Camara, Manar Jallal, Mohamed Khalis, Saad Zbiri, Vincent De Brouwere

**Affiliations:** 1 International School of Public Health, Mohammed VI University of Health Sciences, Bld Mohammed Taïeb Naciri, Commune Hay Hassani, 82 403 Casablanca, Morocco; 2 Laboratory of Public Health, Health Economics and Health Management, Mohammed VI University of Health Sciences, Casablanca, Morocco; 3 Knowledge for Health Policies Centre, Casablanca, Morocco; 4 Department of Public Health, Institute of Tropical Medicine, Antwerp, Belgium; 5 School of Tropical Medicine and Global Health, University of Nagasaki, Nagasaki, Japan

**Keywords:** Community-based health insurance, Mutual health organisations, Membership, Universal health coverage, Health financing, West Africa

## Abstract

**Background:**

In many low-income countries, households bear most of the health care costs. Community-based health insurance (CBHI) schemes have multiplied since the 1990s in West Africa. They have significantly improved their members’ access to health care. However, a large proportion of users are reluctant to subscribe to a local CBHI. Identifying the major factors affecting membership will be useful for improving CBHI coverage. The objective of this research is to obtain a general overview of existing evidence on the determinants of CBHI membership in West Africa.

**Methods:**

A review of studies reporting on the factors determining membership in CBHI schemes in West Africa was conducted using guidelines developed by the Joanna Briggs Institute. Several databases were searched (PubMed, ScienceDirect, Global Health database, Embase, EconLit, Cairn.info, BDPS, Cochrane database and Google Scholar) for relevant articles available by August 15, 2022, with no methodological or linguistic restrictions in electronic databases and grey literature.

**Results:**

The initial literature search resulted in 1611 studies, and 10 studies were identified by other sources. After eliminating duplicates, we reviewed the titles of the remaining 1275 studies and excluded 1080 irrelevant studies based on title and 124 studies based on abstracts. Of the 71 full texts assessed for eligibility, 32 additional papers were excluded (not relevant, outside West Africa, poorly described results) and finally 39 studies were included in the synthesis. Factors that negatively affect CBHI membership include advanced age, low education, low household income, poor quality of care, lack of trust in providers and remoteness, rules considered too strict or inappropriate, low trust in administrators and inadequate information campaign.

**Conclusions:**

This study shows many lessons to be learned from a variety of countries and initiatives that could make CBHI an effective tool for increasing access to quality health care in order to achieve universal health coverage. Coverage through CBHI schemes could be improved through communication, improved education and targeted financial support.

## Introduction

Over the last few decades, most low- and middle-income countries (LMICs) have been confronted with sustainability issues related to their health care systems. In these countries, out-of-pocket payments account for a large proportion of the health care expenditures. This contrasts with European countries which have moved towards mandatory social health insurance systems, thereby reducing the proportion of out-of-pocket payments [1, 2].

Each year, approximately 44 million households around the world -more than 150 million people- face very important health care expenditures and over 100 million people fall into poverty due to health care costs [3].

To overcome these difficulties, community-based health insurance (CBHI) systems have emerged in Sub-Saharan Africa. A CBHI is a non-profit organisation governed by its members, based on the principles of solidarity and mutual aid between individuals who participate freely and voluntarily. It aims to improve access to health care for the population by sharing the financial risks of illness among its members [4, 5]. Despite the efforts made to develop CBHI schemes, the low levels of membership, both initial enrolment and renewal of membership, continue to threaten their financial viability while encountering a multitude of obstacles to their development [5, 6].

Among the various challenges facing CBHI schemes, enrolment currently appears to be the predominant issue [5–7]. Although the mutualist movement is growing steadily in West Africa, the coverage of the target population rarely exceeds 30% [7–10]. One thing is clear everywhere: the membership rates of CBHI schemes remain relatively low, sometimes even calling into question the very viability of the organisation [5, 11].

To try to understand this low participation rate of the population, one can question the economic theory of health insurance by trying to define its essential parameters for the specific context of West African countries. However, our main objective here is at another level and is based on the following observation: over the past decade, several empirical studies have been conducted, albeit with widely varied methodologies, on factors that may explain the decision to participate or not in a CBHI in West Africa. In this context, we would like to see if, beyond this great methodological heterogeneity, it is nevertheless possible to identify some convergent results that could enlighten public and private actors in this field.

Although there are already a few reviews on the determinants of CBHI membership in LMICs [8, 9, 11, 12], this review has several advantages. Firstly, it aims to update existing data by considering a broad time period up to 2022. Second, it focuses on the West African region. This group of countries, different from other LMICs, shares particular socioeconomic and political characteristics that influence their health care financing and systems. These countries suffer from economic inefficiencies and weak public governance, and their populations are largely rural with low financial capacity. Their health sectors are often underfunded and the quality of public health services offered is limited [1–4].

## Methods

### Research strategy and data source

We conducted a review of studies reporting on the factors explaining the persistence of the low rate of CBHI membership in West Africa using the guidelines for conducting scoping reviews developed by the Joanna Briggs Institute [13].

We developed a research strategy for relevant studies published between January 1, 2000 and August 15, 2022, without methodological and language restrictions, in the electronic databases PubMed, ScienceDirect, Global Health database, Embase, EconLit, Cairn.info, BDPS, Cochrane database and Google Scholar, using combinations of the following keywords: [“determinant” OR “factor” OR “driver”] AND [“community insurance” OR “community mutual” OR “mutual health”] AND [“membership” OR “enrolment” OR “subscription” OR “participation” OR “adherence” OR “retention” OR “take-up” OR “uptake” OR “demand” OR “drop-out”] AND [“West Africa”].

To make the research comprehensive and to identify additional articles, we sought additional sources and conducted searches in each of the West African countries: Benin, Burkina Faso, Cape Verde, Côte d’Ivoire, Gambia, Guinea, Guinea-Bissau, Liberia, Mali, Mauritania, Niger, Nigeria, Senegal, Sierra Leone and Togo. Ghana was excluded as this country has a national health insurance which is different from the CBHI scheme [6]. We completed the demographic, health and economic data based on the websites of the World Health Organisation (WHO), the official NHIS of the countries of interest and the World Bank. Then we applied the snowball method which allowed us to add relevant articles by reviewing the references of all the studies selected in the previous step.

### Inclusion and exclusion criteria

The review included studies published between January 1, 2000 and August 15, 2022 and articles that reported on the determinants of membership in CBHI in West Africa.

Articles for which full text was not available and those that were clearly not relevant to the topic were excluded.

### Selection of studies

We identified eligible articles using the PRISMA flow chart. The first and second authors independently reviewed all titles and abstracts identified by the research. Full texts of all potentially eligible articles were retrieved and reviewed for inclusion in this review according to the inclusion criteria.

Zotero reference manager software was used to organise and detect duplicate references (www.zotero.org). After removing duplicates, titles and abstracts of all articles were independently reviewed for eligibility, relevance and compliance with inclusion and exclusion criteria by two of the authors (KKC and AMC).

### Data and item extraction

For the included studies, we independently extracted information on study characteristics (authors, title, year of publication, country, research method, sample size and determinants of membership and non-membership). This information was summarized in a spreadsheet, and an evidence table was generated to describe and summarize the characteristics of the included studies (Table 1).

**Table 1:** Determinants of membership and non-membership in community-based health insurance (CBHI) in West Africa

Article	Country	Method	Membership factors	Non-membership factors
Seck et al. [14]	Senegal	Retrospective cross- sectional study Quantitative approach Sample: 912 households	*Individual characteristics* Higher literacy levels Household size less than ten members *Determinants of care benefits* Short distance to a conventional health training	*Individual characteristics* Rural environment Large household size Low literacy level of the head of the household Over 60 years old *Financial capacity* Low-income level *Determinants of care benefits* Poor perception of quality of care High distance from the health care facility
Turcotte-Tremblay et al. [15]	Benin	Multiple case study Qualitative approach Sample: 23 semi-structured interviews, two focus groups and 15 unstructured interviews	*Financial capacity* Flexibility of payment *Determinants specific to CBHI* Raising awareness through door-to-door outreach Government involvement	*Individual characteristics* Low level of education *Determinants of care benefits* Poor quality of health care Negative behaviour of health care professionals *Determinants specific to CBHI* Ignorance of the existence and benefits of CBHI organisations Lack of current knowledge about strategies Lack of public confidence Poor communication Unsuitable payment method Lack of information
Onwujekwe et al. [16]	Nigeria	Cross-sectional study Quantitative approach Sample: 3070 randomly selected households	*Determinants of care benefits* Improving access to good quality health services Urban area Formal education	*Individual characteristics* Low level of education Rural area *Determinants specific to CBHI* Distrust and cynicism about the success of the program *Financial capacity* Poor households *Determinants of care benefits* Geographical distance
Odeyemi [17]	Nigeria	Literature review Sample: 26 included studies	*Financial capacity* Wealth Financial grant from the government *Determinants specific to CBHI* Committing to providing private health insurance and services to low- and moderate-income families Providing cardiovascular disease prevention care in a low-resource environment	*Individual characteristics* Muslims (believed that CBHI was for the Christians) Low level of education *Financial capacity* Low-income level Lack of financial resources *Determinants specific to CBHI* Inadequate financial support for unrealistic enrolment requirements Inability to tailor the implementation to a specific domain Insufficient community involvement Lack of confidence in the program and its management
Smith et al. [18]	Mali, Senegal (and Ghana)	Cross-sectional evaluation study Mixed approach (qualitative and quantitative) Samples: 40 households (qualitative) and 1080 households (quantitative) in Senegal, 2280 households (quantitative) in Mali and 1806 households (quantitative) in Ghana	*Individual characteristics* Catholics Senofo ethnicity Older women Higher level of formal education of the head of the household Higher socioeconomic status *Determinants of care benefits* Modern health care facility	*Individual characteristics* Low level of education Low household standard of living Rural area *Financial capacity* Poverty *Determinants of care benefits* Quality of care Distance *Determinants specific to CBHI* Lack of knowledge about the existence of CBHI
De Allegri et al. [19]	Burkina Faso	Case-control study Quantitative approach Sample:154 enrolled households (cases) and 393 non-enrolled households (controls)	*Individual characteristics* Higher level of education Higher proportion of children living in the household	*Individual characteristics* Bwaba ethnic group Lower social status Low level of education Negative perception of the adequacy of traditional care *Determinants of care benefits* Greater distance from the health care facility
Gnawali et al. [20]	Burkina Faso	Case study Quantitative approach Sample: 1309 households	*Individual characteristics* Membership in any other risk-sharing network Number of children under five years of age	*Individual characteristics* Low level of education of the head of the household Larger households Young people Bwaba ethnic group *Financial capacity* Poor
Chankova et al. [21]	Mali, Senegal (and Ghana)	Cross-country study Quantitative approach Sample: 10,547 households (Mali), 9226 (Senegal) and 9553 households (Ghana)	*Individual characteristics* Households headed by women Higher household education Higher household economic status	
Dong et al. [22]	Burkina Faso	Household survey Quantitative approach Sample: 756 rural and 553 urban households		*Individual characteristics* Female household head Higher age or lower education of household head Fewer children or elderly in household *Determinants of care benefits* Lower number of illness episodes in the past three months Poor perceived health care quality Less seeking care in the past month
Gankpe et al. [23]	Benin	Cross-sectional survey Qualitative approach Sample: 50 patients from three health centres (interviews and focus groups)	*Individual characteristics* Family protection from the risk of illness *Determinants of care benefits* Receiving health care benefits in case of illness	*Individual characteristics* Seniors Large family size Fon ethnic group Woman *Financial capacity* Monthly income less than 30 000 CFA franc *Determinants of care benefits* Contribution for CBHI perceived as a waste in the absence of illness *Determinants specific to CBHI* Lack of trust in management committees Distrust of CBHI leaders Ignorance of the very existence of CBHI Lack of knowledge of how CBHI work Lack of knowledge of the benefits offered
Lyalomhe et al. [24]	Nigeria	Cross-sectional research Quantitative research approach Sample: 372 respondents	*Individual characteristics* Youth and middle-aged persons Females Married people *Determinants of care benefits* Higher use of hospitalization care	
Jütting et al. [25]	Senegal	Cross-sectional study Mixed approach (qualitative and quantitative) Sample: 346 randomly selected households (2860 people)	*Individual characteristics* Better financial protection against the risk of hospitalization Sedentary of certain ethnic groups (Wolofs) *Determinants of care benefits* Short distance to the hospital Good operation of the system	*Individual characteristics* Mobility of certain ethnic groups (Fulani) *Financial capacity* Poor households *Determinants of care benefits* Inefficiency of health services
Franco et al. [26]	Mali	Case-control study Sample: 817 member households and 787 non-member households	*Determinants of care benefits* Closer distance to the conventional health care facility	*Individual characteristics* Large household size Residence far from a health care facility The majority ethnic origin (Bambara) *Financial capacity* Very low-income level
Jütting et al. [27]	Senegal	Household survey data Quantitative analysis Sample: 346 households	*Individual characteristics* Ethnic group of Wolof Christian Village characteristics *Financial capacity* Higher household income	
Criel et al. [28]	Guinea	Cross-sectional study Mixed approach (qualitative and quantitative) Sample: 12 focus groups of 8–12 respondents (137 participants)	*Individual characteristics* Prediction of future illness Preservation of health Guarantee of health *Financial capacity* Avoiding financial difficulties *Determinants specific to CBHI* Guarantee of health insurance Guarantee of health care	*Individual characteristics* Large household members *Financial capacity* Difficulty in paying for all household members at the same time *Determinants of care benefits* Poor quality of care evidenced Lack of good products *Determinants specific to CBHI* Lack of confidence in integrity Lack of management skills
Sagna et al. [29]	Senegal	Literature review and in-depth interviews Qualitative and exploratory approach Sample: 40 heads of households and 12 focus groups	*Financial capacity* Flexibility of payment either in cash or in kind	*Financial capacity* Low-income level Terms of payment of the difficult premium *Determinants of care benefits* Contents of the incomplete benefits package Terms of payment of the difficult premium *Determinants specific to CBHI* Incomprehensible premium amount Lack of availability of transportation Level of co-payment Absence of the guarantee between the agreement with the health institutions and the governance of the CBHI companies
Onwujekwe et al. [30]	Nigeria	Cross-sectional study Mixed approach (qualitative and quantitative) Sample: 3070 selected households	*Individual characteristics* High level of education Gender: men pay more than women *Financial capacity* Poor were in favour of the very low contribution and reported the lowest average amount	*Financial capacity* Low socioeconomic status Poverty *Determinants of care benefits* Remote location of residence
Souares et al. [31]	Burkina Faso	Cross-sectional study Quantitative approach Sample: 7762 households	*Individual characteristics* Level of education *Financial capacity* Subsidies for the poorest Wealth	*Financial capacity* Poverty Difficulty to pay full price to register
Mladovskya et al. [32]	Senegal	Case studies Mixed approach (qualitative and quantitative) Sample: 960 heads of households	*Individual characteristics* Financial protection against the cost of ill health Belonging to an association	*Individual characteristics* Extended families *Financial capacity* Poverty Low share capital
Ndiaye et al. [33]	Guinea	Documentary review and qualitative study	*Individual characteristics* Ensuring access to quality essential emergency obstetric care *Financial capacity* Opportunity to save money	*Financial capacity* Amount of women’s contributions *Determinants of care benefits* Poor quality of care Lack of skills *Determinants specific to CBHI* Low involvement of communities Lack of follow-up Information marks
Onwujekwe et al. [34]	Nigeria	Cross-sectional study Quantitative approach Sample: 971 respondents	*Individual characteristics* Protection against financial risks *Determinants of care benefits* Availability of good quality treatment	*Financial capacity* Unavailability of funds *Determinants of care benefits* Too poorly equipped provider facility *Determinants specific to CBHI* Lack of trust in responsible for managing the health care system
De Allegri et al. [35]	Burkina Faso	Qualitative study Sample: 32 households	*Individual characteristics* Avoiding catastrophic spending Guarantee of the treatment when sick	*Financial capacity* Lack of financial means *Determinants of care benefits* Distance Absence of a doctor in the village Dissatisfaction with the quality of care *Determinants specific to CBHI* Lack of trust Lack of knowledge and understanding Institutional rigidity
Ridde et al. [36]	Benin	Exploratory study Qualitative approach Sample: 20 groups and 29 individuals		*Financial capacity* Problems in being able to pay the premiums *Determinants of care benefits* Lack of trust in health care professionals *Determinants specific to CBHI* Lack of information
Ridde et al. [37]	Benin	Cross-sectional study Qualitative approach Individual interviews with members and non-members	*Determinants specific to CBHI* Information that caregivers can no longer cause them harm Impossible to do drug fraud	*Financial capacity* Lack of financial means Inability to pay the required premiums for membership *Determinants specific to CBHI* Lack of information about the risks covered
Alenda-Demoutiez et al. [38]	Senegal	Case study Qualitative approach Sample: 66 semi-structured interviews	*Individual characteristics* Facilitation of access to care Strengthening of social cohesion	*Individual characteristics* Low level of education *Financial capacity* Poverty *Determinants specific to CBHI* Lack of information Lack of awareness
Bastin [39]	Benin	Cross-sectional study Qualitative approach (focus group) Sample: 29 groups	*Financial capacity* Reduction of poverty	*Individual characteristics* Large family *Financial capacity* Lack of financial resources *Determinants of care benefits* Bad reception Poor quality of care *Determinants specific to CBHI* Lack of information
Bonan et al. [40]	Senegal	Cross-sectional study Quantitative approach Sample: 360 households	*Financial capacity* Willingness to pay	
Koloma [41]	Burkina Faso	Cross-sectional study Qualitative approach Sample: 112 households	*Financial capacity* Protection against potential disease risks Reduction of monthly health expenses Improvement of well-being	*Individual characteristics* Large family Low level of education
Sow et al. [42]	Senegal	Cross-sectional study Quantitative approach Sample: 392 patients	*Individual characteristics* Perception Presence of an elderly person in the household Household size *Determinants of care benefits* Pyramid medical institution *Determinants specific to CBHI* Confidence in CBHI	
Mladovsky [43]	Senegal	Household cross-sectional survey Quantitative analysis Sample: 382 members and ex-members	*Determinants specific to CBHI* Active mode of participation in the CBHI including training; voting, participating in a general assembly, awareness raising/information dissemination and informal discussions/spontaneously helping; perceived trustworthiness of the scheme management/president, accountability and being informed of mechanisms of controlling abuse/fraud	*Determinants of care benefits* Perception of poor quality of health services
Bousmah et al. [44]	Senegal	Retrospective cross-sectional study Quantitative approach Sample: 1002 households	*Individual characteristics* Individual risk preference *Determinants specific to CBHI* Health insurance awareness	*Determinants of care benefits* Geographic distance
Waelkens et al. [45]	Mauritania	Case study Retrospective study over a ten-year period (2003 to 2012) Qualitative approach		*Determinants specific to CBHI* Ineffective procedures for premium collection Complicated procedures for proving entitlement when seeking care Poor understanding of multiple and complicated rules and regulation Disinformation by recruiters with the aim to register high numbers Distrust of members resulting of insufficient information, disinformation and mismanagement of funds No perception of belonging and ownership by members Poor performance of delegates in positions of responsibility, who had expected personal rewards Exclusion of the poorest and large households; disinterest of wealthier households Inaction of scheme leaders when faced with unexpected problems
De Allegri et al. [46]	Burkina Faso	Cross-country study Qualitative approach Sample: 32 households	*Determinants specific to CBHI* CBHI elements that match consumers’ needs and expectations in relation to their preference for the unit of enrolment, the premium level and the payment modalities, the benefit package, the health service provider network and the managerial structure	
Mladovsky et al. [47]	Senegal	Three case studies Qualitative evidence Sample: 64 CBHI stakeholders	*Determinants specific to CBHI* Subsidising the salaries of CBHI scheme staff High sustainable internal and external governance structures through CBHI federations CBHI resonance with local values concerning four dimensions of solidarity (health risk, vertical equity, scale and source) Increased transparency in national policy Increased leaders’ negotiating power vis-à-vis health service providers	
Rouyard et al. [48]	Senegal	Retrospective study Quantitative analysis Sample: 676 CBHIs	*Determinants specific to CBHI* Higher operational capacity Presence of a salaried manager at the CBHI level Stronger cooperation between CBHIs and local health posts CBHIs located within a health facility enrolling fewer poor members	
Bocoum et al. [49]	Burkina Faso	Randomized experiment Quantitative approach Sample: 2000 households	*Determinants specific to CBHI* CBHI information increases understanding in poorer households and in households with literate heads	
Bonan et al. [50]	Senegal	Randomized controlled trial Quantitative approach Sample: 360 households	*Determinants specific to CBHI* Insurance marketing treatments	
Cofie et al. [51]	Burkina Faso	Cross-country study Quantitative and qualitative approach Sample: 250 households	*Determinants specific to CBHI* Information, education and communication campaign	
Yusuf et al. [52]	Nigeria	Cross-country study Qualitative approach Sample: 419 respondents	*Individual characteristics* Health insurance-related knowledge and attitudes	

After screening and obtaining full texts of the selected articles, we examined each publication for information on the characteristics of the determinants of membership and non-membership in CBHI, with a particular focus on recurring observations in the different countries. The observations were used to formulate recommendations for policy decisions likely to improve membership rates of CBHI schemes in West Africa. Any discrepancies in the selection and extraction process were resolved by discussion, if necessary, with two other authors (SZ and VDB).

## Results

**Fig. 1:**
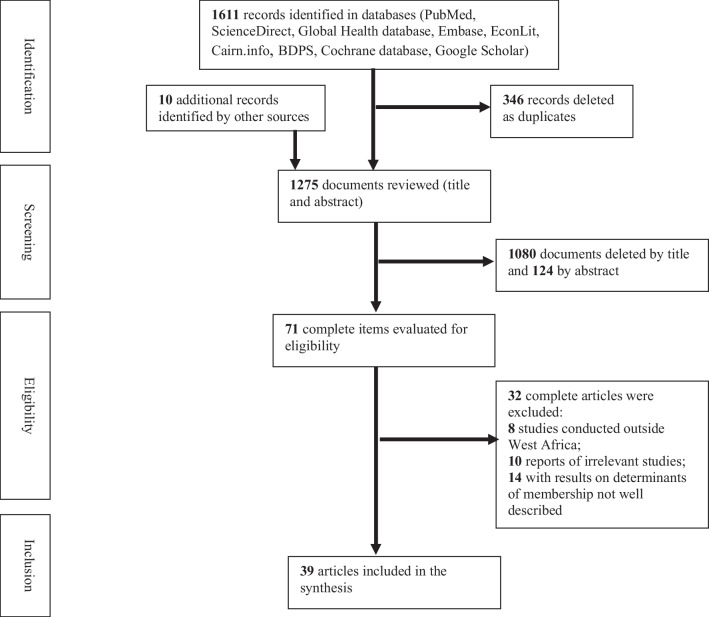
PRISMA flow chart of the study selection process for examining the determinants of membership in community-based health insurance (CBHI) in West Africa (2000–2022)

The initial literature search yielded 1611 studies and ten additional studies were identified by other sources. After eliminating duplicates, we reviewed the titles of the remaining 1275 studies and excluded 1080 irrelevant studies by title and 124 studies based on abstracts. Of the 71 full texts assessed for eligibility, 32 additional papers were excluded (not relevant, outside West Africa, poorly described results) and finally 39 studies were included in the synthesis (Fig. 1).

### Key determinants of CBHI membership

Most articles were based on cross-sectional household surveys, case studies and interviews. Of the 39 studies included in the review, 17 were quantitative, 15 were qualitative, six were mixed-method studies (quantitative and qualitative approaches) and one study was exclusively a literature review. The sample sizes of the studies ranged from 23 to 29 326 households for the quantitative and mixed-method studies. The 39 studies that met our inclusion criteria were published between 2003 and 2022, mostly within the last ten years. The studies were conducted in only seven West African countries: 13 in Senegal, nine in Burkina Faso, six in Nigeria, five in Benin, two in Guinea, one in Mali, one in Mauritania and two multi-country studies (Table 1). The majority of the studies were community-based, mostly rural, except for the literature review studies. Other studies were conducted in healthcare facilities, health centres or hospitals.

The main factors of membership in CBHI schemes were identified and presented as follows: individual characteristics (age, level of education, ethnicity and religion); household socioeconomic characteristics (household financial capacity, household size, associative history, target group cohesion and therapeutic use); determinants related to healthcare services (quality of healthcare, trust in the skills of healthcare providers and geographical distance) and parameters specific to CBHI (membership procedures, services offered to members, trust in the CBHI schemes, information and understanding).

### Individual characteristics

Nine studies show that membership appears to be related to certain individual characteristics, including the level of education that influences participation in the CBHI schemes. Literate people are more likely to participate in a CBHI scheme than illiterate people [14–22]. Some of our studies reported that people aged 65 and over are more economically and socially excluded from the community and therefore have more difficulty being part of CBHI [14, 22–24]. Seven empirical studies found greater affiliation of certain ethnic groups to CBHI schemes [18–20, 23, 25–27]. In a study in Senegal, the Peulh/Fulani ethnic group was less likely to participate in the program probably because of their nomadic lifestyle [25]. Religion, although not widely considered as a membership factor in the selected studies, could also influence household membership in a community-based health financing system. In the early 2000s, Muslims were found to be much less likely than Christians to participate because they mistakenly believed that the projects were open only to Christians [17, 18, 27].

### Socioeconomic characteristics of households

#### Financial capacity of households

The household income level appears to be an essential parameter for membership and most studies indicate that a low capacity to contribute is a major obstacle to participate in a community health insurance system. Lack of financial resources is often the first reason given by both members and non-members to explain low participation in CBHI [14, 16–18, 22, 23, 25, 26, 28–39]. Several studies have shown that the socioeconomic level of members is higher than that of non-members [17, 18, 21, 25, 30, 31]. Although respondents most often consider the amount of the individual contribution to be fair or affordable (especially compared to the costs of not participating), many cannot afford to pay the contributions for all the members of the household. Households’ standard of living is summarized by three separate indicators: income, annual expenditures and perception of their financial capacity relative to others. This enables us to establish that the poorest households are much less represented in CBHI than the better-off households. Most members consider that CBHI schemes offer better financial protection [14, 16–18, 22, 23, 25–40].

#### Household size

Regarding household size, some empirical work presents results that state the greater difficulty of large households to participate in a health risk pooling system. Although these families are not considered among the poorest in the community, they are generally unable to bear the contributions for all the members of the household [14, 22, 23, 26, 28, 39, 41, 42].

#### Association history, target group cohesion, therapeutic use and health perceptions

The existence of informal risk-sharing associations appears to facilitate the establishment of a community health insurance scheme; the involvement of community leaders positively influences membership. One study shows that 41% of CBHI members say they signed up on the advice of an influential person and research in Burkina Faso indicates that members of a community health insurance system have a more negative perception of traditional healthcare than non-members, often judging it to be poor or ineffective [19, 20, 30, 32, 42].

### Determinants of caregiving

The role played by healthcare providers in the dynamics of CBHI memberships can be considered from three angles: the quality of the healthcare, the population’s trust in the skills of healthcare providers and the geographical proximity of the healthcare centres under agreement.

#### Quality of healthcare

Among the included studies, many showed that factors related to healthcare quality and provider relationships were important factors in CBHI membership. On the one hand, poor quality of the care provided, including patient reception, prescription and availability of drugs, effectiveness and duration of the treatment, negatively influenced membership in a CBHI scheme. On the other hand, doubts about the organisation’s ability to improve healthcare quality also discouraged membership [14–16, 18, 22, 28, 33, 35, 39, 43].

The quality of the healthcare provided is an encouraging factor, even a membership condition and may also be a consequence of membership through members’ pressure to improve quality [14, 18, 26, 28, 30, 35].

#### Confidence in the skills of providers

In addition to user dissatisfaction with the attitude of healthcare providers towards patients, empirical research attests of distrust in the skills of healthcare workers. This scepticism about health workers’ competence reinforces the lack of trust in health workers, which negatively influences membership. Thus, trust in health workers is based above all on their professional experience and ability to listen to and inform the patient [15, 22, 23, 25, 29, 33, 35, 36].

#### Geographical distance

The results of surveys on the distance to health centres highlight geographic distance as a significant barrier to membership and even as a reason for disaffiliation [14, 16, 18, 19, 25, 26, 30, 34, 35, 44].

### Determinants specific to CBHI schemes

Regarding membership, the community health insurance company as such could be examined according to three aspects: first, the membership modalities and the services it offers to its members, second, the trust that the communities place in it and third, the way in which it sets up and invests in the information and sensitization of the target populations.

#### Terms of membership and benefits offered to members

Memberships, dues and premiums, as mentioned above, are considered acceptable by the communities, despite the lack of financial resources of many of them. The frequency of payment of the membership fee seems to influence membership: it appears that the obligation to pay the membership premium and/or the annual fees at once is a major obstacle, particularly for large families [15, 29, 35, 45, 46]. The lack of coverage of certain benefits is also an obstacle to membership for some individuals. Finally, the rigidity of certain internal rules established by the CBHI scheme also hinders membership. For example, one study indicates that there is a problem of affordability for many poor and/or large families who cannot gather enough money to pay for membership of all of the household members in one go. Although it is a measure against adverse selection, this rule is perceived by communities as too rigid and inappropriate [15, 29, 35, 45–47].

#### Trust in the CBHI scheme

Confidence in the CBHI scheme also influences the decision to participate or not. Studies highlighted two dimensions of the target population’s confidence in CBHI: on the one hand, confidence in the management of the system which depends on the skills and integrity of the CBHI managers, and on the other hand, confidence in the ability of the CBHI scheme to achieve its stated objectives. The target populations frequently adopt a cautious attitude at the launch of a CBHI scheme, preferring to observe before participating [15–17, 23, 28, 34, 35, 43, 45–48]. Other studies indicate that doubts about the honesty of the CBHI leaders or previous experiences with embezzlement may have a negative impact on membership [19, 35, 42, 43, 45–48].

#### Misunderstanding and lack of information

Inadequacy of communication is a factor influencing membership in CBHI schemes. Information to be communicated concerns the very existence of the CBHI system, knowledge of how the CBHI scheme functions and knowledge of the benefits offered by the CBHI scheme. The transmission of information is said to be deficient because the awareness campaign is not very attractive or not adapted to illiterate populations. This indicates that non-members have a limited understanding of community health principles [15, 17, 18, 23, 33, 35–39, 43–45, 49–52].

## Discussion

This review aims to identify the factors influencing membership in CBHI schemes in West Africa. The synthesis of the studies made it possible to identify the factors that influence the decision to participate or not in a CBHI. These factors are mainly related to individual characteristics (age, level of education, ethnicity and religion), household size, household financial capacity, associative history, quality of health care, confidence in the skills of healthcare providers, geographical distance, membership modalities, benefits offered to members, confidence in the CBHI, information and understanding. As CBHI initial enrolment, membership renewal and drop-out determinant factors are relatively the same, we have chosen to analyse these membership factors together.

This study suggests that the people potentially interested by CBHI schemes are those who are middle-aged. A similar finding was reported by Fadlallah et al. in a systematic review [53]: in low-income countries, nine out of 51 quantitative studies suggested a positive correlation with older age (i.e., 36 years and older, on average). Regarding larger families, five out of 51 studies confirmed that larger households were less likely to participate in a CBHI. The non-membership of the elderly is likely to be part of a broader lack of social protection for this group, with, for example, less than 7% of people aged 60 and over receiving social protection in Benin [23].

Low education was found to be a barrier in ten of the included studies. These results are consistent with those reported by previously-published reviews in Sub-Saharan Africa, which showed that the higher the level of education, the greater the likelihood of participating in a CBHI, whether measured in terms of years of schooling or literacy skills [8, 9, 11].

Factors related to healthcare and relationships with healthcare providers are also important determinants of membership in a CBHI. On the one hand, poor quality of care negatively influences membership. On the other hand, people’s scepticism related to the skills of health personnel reinforces their lack of confidence in them. In addition, doubts about the organisation’s ability to improve the quality of care also contributes to non-membership. The reasons for the lack of trust in the CBHI may also be due to previous negative experiences or to suspicion of dishonesty towards the CBHI managers. These observations call for special attention to be paid to contracting modes between CBHI schemes and healthcare services. For start-up CBHI organisations, or more generally those of limited size, there is a high probability that they will not be able to influence the practices of healthcare facilities and workers. Several experiences suggest, however, that this type of situation is not inevitable. For example, when a hospital is actively involved in, or even initiates, community insurance systems, access to care, quality of care and patient confidence are significantly improved as shown in the work of Fadlallah et al. and Jütting and Tine on mutualist systems in the Thiès region of Senegal [53, 54].

Distance between the place of residence and the healthcare facility constitute a known barrier to participating in a CBHI scheme [8, 9, 11, 12]. There is an exception in Burkina Faso. De Allegri et al. showed that the distance separating mutualists from the healthcare facility was not a barrier to participate to the CBHI [35]. Membership rates were higher in the communities furthest from the health centre. This surprising result was explained by the intense promotion campaigns carried out among the most distant populations and by the fact that the CBHI scheme covers the cost of transport.

The results of our review show that the lack of information and understanding by the population can be an obstacle for CBHI membership and similar results were previously reported in different reviews [8, 9, 12, 53]. It is very often through radio and newspapers that messages are delivered. The information does not reach the relevant targets because the majority of the population, especially the most disadvantaged, do not have access to these media. This explains why some articles reported that door-to-door activities with elected members of CBHI schemes were the most effective initiative for increasing membership [55]. Communication strategies are often lacking and deserve more investment to make population aware of CBHI.

There is broad consensus among the included studies that lack of financial resources remains the main reason stated by households for not participating in a CBHI. This is similar to the mixed results on economic status found by Fadlallah et al. and Ahmad et al. in India [53, 56]. Consequently, the poorest households cannot afford paying a membership and are therefore excluded [57].

CBHI schemes that are more flexible with the collection and timing of contributions and with the membership rules would be more likely to attract new members. Jütting and Tine broke down this process into several stages during which the perception of the current and expected costs and benefits of insurance vary according to factors linked to both the situation of the individuals and the general environment [54].

In most West African countries, there are tens, sometimes hundreds different small local initiatives which positively reflect a local dynamic but at the same time may be the reason of the failure. Indeed, CBHI schemes are based on solidarity among the members (risk pooling) and logically the number of members matters. Some countries, however, managed to succeed. Examples of Ghana, Rwanda (the only country in sub-Saharan Africa to achieve a coverage above 90% [58, 59]) or India are interesting case studies. The key to Rwanda’s success in implementing the health insurance program appears to be a strong societal consensus on equal access to healthcare with financial protection, crucially supported by the government through investments in the health sector, effective legislation to provide basic care to the uninsured and an intensive national program [58, 59].

In India, most CBHI programs are run by nongovernmental organisations with considerable organisational diversity [60, 61]. Strategic procurement and effective monitoring were provided, and financial sustainability was increased by merging smaller systems. In particular, the Indian government has created a more favourable policy environment by giving legal recognition to CBHI programs and providing grants to the poor [61, 62]. These programs have been rapidly successful and reflected a trend in Asian countries to move away from informal coverage and to integrate health insurance programs into regulated government frameworks. Although the health insurance landscape in Asia differs somewhat from that in West Africa, some common patterns and lessons can be drawn.

Senegal is a strange case because it implemented the known ingredients of success but did not reach the expected coverage. The government launched in 2013 a universal health coverage initiative based on local CBHI organisations that reached 676 in 2016. The national agency standardized the benefit package and insurance premium, the government subsidized 50% of the premium for all enrolees and 100% for the poor and disabled people, and the rest of the funds from the premiums are pooled to cover health care and drugs provided or prescribed by referral hospitals [63]. According to Bousmah et al., the problem was linked to the low demand for health insurance due to limited access to information about available schemes [44].

So far, CBHI, without a strong support from governments and international aid, seems ineffective to reach universal health coverage. However, CBHI may have a role in increasing uptake of quality care and promoting accountability of healthcare providers.

The originality of our study is to review factors of CBHI membership and non- membership in West Africa and it was conducted with respect to research standards of scoping review. However, despite our best effort to review all available recent evidence available, this study has limitations. First, we found data in only seven of the 15 West African countries, while several other West African countries have previously launched similar insurance programs. Second, the quality of the included studies was not considered. Lastly, as it was not a systematic review and meta-analysis, the statistical magnitude of the different determinants of CBHI membership and non-membership were not compared.

## Conclusions

This study shows many lessons that can be learned from a variety of countries and initiatives that could make CBHI an effective tool for expanding access to healthcare. Coverage through CBHI schemes could be improved through policies that include closer integration of the informal and formal sectors within the existing national health insurance systems, with increasing beneficiary participation in program design and management, improved communication and education, increased public and private financing of healthcare and targeted financial assistance. By making membership mandatory, more people could benefit from social protection. These results are likely to be instructive for government policymakers in achieving universal health coverage goals and improving health outcomes.

## Data Availability

Not applicable.
